# Overview of the improvement of the ring-stage survival assay-a novel phenotypic assay for the detection of artemisinin-resistant *Plasmodium falciparum*

**DOI:** 10.24272/j.issn.2095-8137.2017.075

**Published:** 2017-11-18

**Authors:** Jie Zhang, Guo-Hua Feng, Chun-Yan Zou, Pin-Can Su, Huai-E Liu, Zhao-Qing Yang

**Affiliations:** ^1^Department of Pathogen Biology and Immunology, Kunming Medical University, Kunming Yunnan 650500, China; ^2^Biomedical Engineering Research Center, Kunming Medical University, Kunming Yunnan 650500, China; ^3^Guangxi Zhuang Autonomous Region People's Hospital, Nanning Guangxi 530021, China; ^4^Transfusion Medicine Research Department, Yunnan Kunming Blood Center, Kunming Yunnan 650500, China; ^5^Department of Infectious Diseases, The First Affiliated Hospital, Kunming Medical University, Kunming Yunnan 650032, China

**Keywords:** Malaria, Artemisinin, Resistant phenotype, Ring-stage survival assay, Improvement, Application

## Abstract

Artemisinin resistance in *Plasmodium falciparum* threatens the remarkable efficacy of artemisinin-based combination therapies worldwide. Thus, greater insight into the resistance mechanism using monitoring tools is essential. The ring-stage survival assay is used for phenotyping artemisinin-resistance or decreased artemisinin sensitivity. Here, we review the progress of this measurement assay and explore its limitations and potential applications.

## INTRODUCTION

Malaria, which is mainly caused by *Plasmodium falciparum*, is a long-term worldwide public health problem. An estimated 216 million new cases occurred globally in 2016, resulting in 445 000 deaths (WHO, 2017). Despite significant progress in reducing morbidity and mortality rates in many areas of endemicity, drug resistance has become a challenging issue. Since *P. falciparum* developed resistance to chloroquine and sulfadoxine-pyrimethamine, malaria has spread rampantly throughout Asia and Africa over the last several decades (Snow et al., 2001; Trape et al., 1998), posing serious difficulties for its control and elimination.

Originally discovered in China, artemisinin (ART) and its derivatives, including dihydroartemisinin (DHA), artemether, and artesunate, demonstrate high performance, low toxicity, and limited cross-resistance with other antimalarial drugs (Li et al., 1979; Miller & Su, 2011). ART is at the frontline for the treatment and possible cure of malaria (Fairhurst, 2015); however, along with its global application, resistance to ART has developed and increased in many regions. Since its first detection in 2008 (Noedl et al., 2008) and 2009 (Dondorp et al., 2009) in western Cambodia, ART resistance has appeared successively in other countries of the Greater Mekong Subregion, manifesting with a reduced parasite clearance rate or prolonged *in vivo* parasite clearance time following treatment with ART-based combination therapies (ACTs) (Amaratunga et al., 2012; Ashley et al., 2014; Hien et al., 2012; Huang et al., 2015; Kyaw et al., 2013; Phyo et al., 2012). For many decades, Southeast Asia (SEA) has been an epicenter for the evolution of drug-resistant falciparum malaria, and the emergence of ART resistance in SEA is of great concern for the global control of falciparum malaria (Fairhurst, 2015).

## RING-STAGE SURVIVAL ASSAY

Hidden within ART-resistant parasites is the ability to remain dormant in the ring stage after exposure to ART, as well as recovery at a rapid rate, resulting in numerous parasites enduring DHA-exposed dormancy (Codd et al., 2011; Teuscher et al., 2010). Due to these special characteristics, despite substantial reductions in the clinical response to ART observed in falciparum malaria, *in vitro* concentrations resulting in 50% growth inhibition in a conventional 48 h exposure assay were relatively low and did not contribute to slow parasite clearance or ACT failure (Dondorp et al., 2009; Saralamba et al., 2011; Woodrow & White, 2017). It is, therefore, necessary to implement rapid and exact monitoring to halt the further spread of ART-resistance.

Hence, the ring-stage survival assay (RSA) was recently established as a new protocol in the surveillance of ART resistance, and can distinguish culture-adapted isolates with fast clearance or slow-clearing rates that can survive pharmacologically relevant doses of ART (Dondorp et al., 2009; Witkowski et al., 2013a). Previous therapeutic efficacy studies have demonstrated a clear correlation between RSA *in vitro* and day 3 parasitemia positivity as well as mutations in the Kelch domain gene (*K13*) associated with resistance (Woodrow & White, 2017; Ariey et al., 2014; Wang et al., 2015; Zhang et al., 2016). Current data have shown RSA to be an important assay for ART resistance *in vitro*.

**Figure 1:**
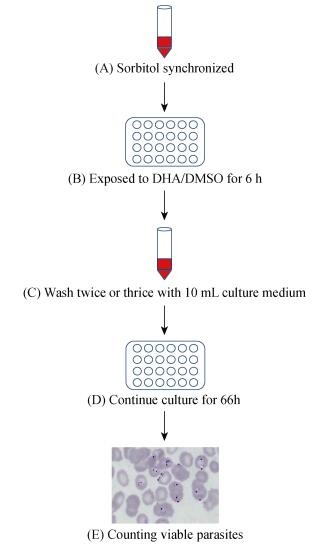
Schematic representation of the ring-stage survival assay *in vitro*

In RSA, young ring-stage parasite cultures (0-3 h), tightly synchronized by 5% sorbitol, are exposed to 700 nmol/L DHA or 0.1% dimethyl sulfoxide (DMSO) as controls for 6 h, then cultivated for 66 h after twice or thrice drug washing. At the end of the assay, survival rates of these isolates are calculated as the ratio under the microscope of viable parasites surviving DHA-induced incubation relative to initial conditions (<a href="http://www.wwarn.org/tools-resources/procedures/ring-stage-survival-assays-rsa-evaluate-vitro-and-ex-vivo-susceptibility" target="_blank">http://www.wwarn.org/tools-resources/procedures/ring-stage-survival-assays-rsa-evaluate-vitro-and-ex-vivo-susceptibility</a>). In general, a ≥1% survival rate is defined as an ART-resistant strain (<xref ref-type="fig" rid="F1-ZoolRes-38-6-317">Figure 1</xref>). 

This method can decrease interference caused by the internal variables of the host, such as organism immunity level, allosteric effects of hemoglobin, and capability to metabolize drugs (Amaratunga et al., 2012; Witkowski et al., 2013a). RSA *in vitro* is proposed to give phenotypic information, thus enabling screening for reduced susceptibility to ART in prolonged clearance parasites (Witkowski et al., 2013b).

## IMPROVED METHODS FOR RSA

While feasible and efficient for the surveillance of ART resistance, the RSA tool has several limitations, including sophisticated Percoll gradient centrifugation, biased assessment of the degree of sorbitol synchronization treatment, and exacting requirements for counting viable parasites (Witkowski et al., 2013a). In Whitney A. Kite's laboratory, two alternative RSA methods have been developed; that is, filtration ring-stage survival assay and sorbitol-only ring-stage survival assay. The first is essentially a filtration process in which the 0-3 h fresh post-invasion rings are obtained after filtering out the merozoites from mature forms. The latter assay performs a repeated step of high synchronization prior to measurement, with the remaining late-stage schizonts typically removed, except for the early rings. Compared with the standard RSA protocol, these modifications have shown a marked increase in phase-specificity as well as less time in culture, fewer lab resources, and lower volume of isolates (Kite et al., 2016). In addition, to limit the inherent variability of microscopic examination, Amaratunga et al. (2014) developed a quick and simple bi-color flow cytometric assay -RSA-2FACS and MitoTracker deep red FM (MTDR) -to accurately quantify observations of viable parasites applied to the RSA. In their study, mitochondrial DNA is readily dyed using the Mito Tracker deep red FM method, allowing for the selection of viable parasites from pyknotic strains (Amaratunga et al., 2014). In addition, Dogovski et al. (2015) suggested direct assessment of the drug-induced growth effects in western Cambodian parasites. For this, the RNA-binding dye SYTO-61, which can distinguish isolates in different stages, was used as a fluorescent marker to determine whether parasites that survived DHA exposure exhibited growth retardation. By comparison with no-drug controls, the decreased SYTO-61 signal in the drug-treated samples exhibited an absolute increase in the number of viable parasites (Dogovski et al., 2015).

## PROSPECT AND APPLICATION OF RSA

The traditional RSA approach was first carried out by Witkowski and colleagues based on sensitivity to DHA exposure at different stages. Their results demonstrated that median ring-stage survival of laboratory lines collected in western Cambodia with slow-clearing infection was 47-fold higher than those with normal ART sensitivity in RSA^0-3h^ (0.23 and 10.88%, respectively), whereas no significant differences were observed in RSA^9-12h^ or trophozoite-stage survival assay (TSA^18-21h^) rates (Witkowski et al., 2013a). In contrast, Cui et al. (2012) demonstrated that the ART resistance phenotype was associated with the dormancy mechanism not only at the development of the ring stage, but also in trophozoites and schizonts (Cui et al., 2012). Thus, the different consequences involved in the characteristics of this phenotype require additional empirical evidence. Cooper et al. (2015) documented that DHA susceptibility using the standard RSA based on the IC50 value for ART failed to clarify resistance in *P. falciparum* parasites from Kampala, Uganda, with the parasitemia of almost all isolates dropping to a much lower level (≤0.025%) after the 72-h assay, revealing no sign of ART resistance. Susceptibility to ART in Cameroonian isolates was also identified using *ex-vivo* RSA, with the DHA-treated cultures showing almost no healthy-appearing parasites (median survival rate=0.49%, IQR=0% to 1.3%) (Menard et al., 2016). However, reduced ART drug *in-vitro* sensitivity of parasites from the China-Myanmar border was reported after assessment of early ring-stage survival by comparing 34 clinical isolates with the 3D7 reference standard strain (Zhang et al., 2016).

The RSA^0-3h^ assay was recently developed to test ART resistance for *P. falciparum* isolates. This assay has been subsequently improved in terms of simplicity and practicality. Thus, the growing availability of RSA will increase the convenience and ease of investigating ART responses in laboratory testing.
